# Threonine 57 is required for the post-translational activation of *Escherichia coli* aspartate α-decarboxylase

**DOI:** 10.1107/S1399004713034275

**Published:** 2014-03-21

**Authors:** Michael E. Webb, Briony A. Yorke, Tom Kershaw, Sarah Lovelock, Carina M. C. Lobley, Mairi L. Kilkenny, Alison G. Smith, Tom L. Blundell, Arwen R. Pearson, Chris Abell

**Affiliations:** a Astbury Centre for Structural Molecular Biology, University of Leeds, Leeds LS2 9JT, England; b School of Chemistry, University of Leeds, Leeds LS2 9JT, England; c Department of Biochemistry, University of Cambridge, Tennis Court Road, Cambridge CB2 1QW, England; d Department of Plant Sciences, University of Cambridge, Downing Street, Cambridge CB2 3EA, England; e University Chemical Laboratory, University of Cambridge, Lensfield Road, Cambridge CB2 1EW, England

**Keywords:** aspartate decarboxylase, post-translational modification, amino-acid-derived cofactors, pyruvoyl-dependent

## Abstract

Aspartate α-decarboxylase is a pyruvoyl-dependent decarboxylase required for the production of β-alanine in the bacterial pantothenate (vitamin B_5_) biosynthesis pathway. The pyruvoyl group is formed *via* the intramolecular rearrangement of a serine residue to generate a backbone ester intermediate which is cleaved to generate an N-terminal pyruvoyl group. Site-directed mutagenesis of residues adjacent to the active site, including Tyr22, Thr57 and Tyr58, reveals that only mutation of Thr57 leads to changes in the degree of post-translational activation. The crystal structure of the site-directed mutant T57V is consistent with a non-rearranged backbone, supporting the hypothesis that Thr57 is required for the formation of the ester intermediate in activation.

## Abstract 2

Threonine 57 is identified as the key residue required for the post-translational activation of *E. coli* aspartate decarboxylase. The crystal structure of the site-directed mutant T57V is reported.

## 1. Introduction   

**Figure 1:**
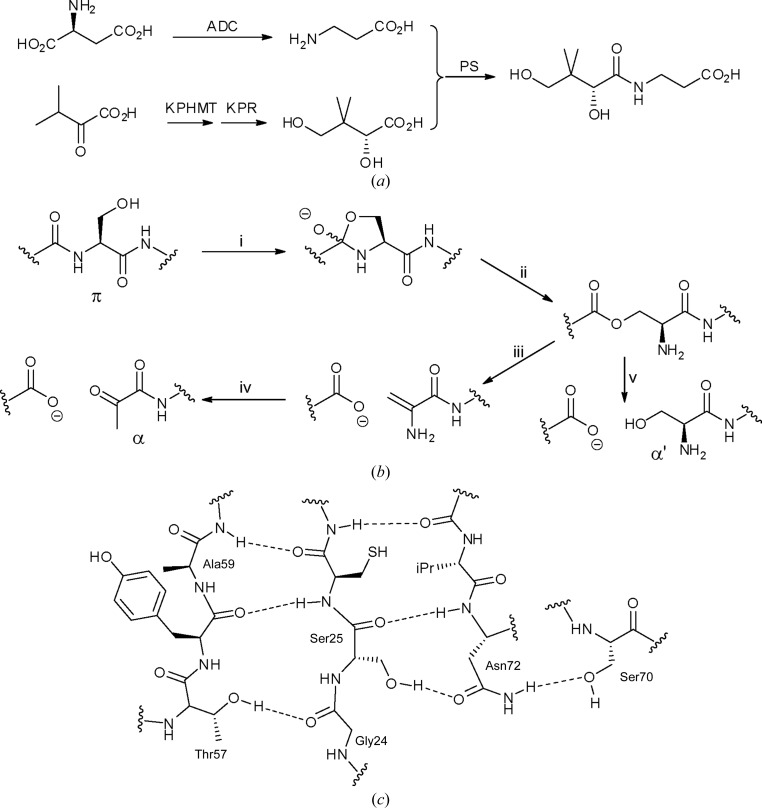
(*a*) Biosynthetic pathway to pantothenate in bacteria. β-Alanine is synthesized *via* the decarboxylation of l-aspartate by aspartate decarboxylase (ADC). Pantothenate synthetase (PS) catalyses the ATP-dependent ligation of β-alanine with d-­pantoate to form pantothenate. Pantoate is formed from α-ketoisovalerate *via* hydroxymethylation by ketopantoate hydroxymethyltransferase (KPHMT) and reduction by ketopantoate reductase (KPR). (*b*) Pathway for the formation of the pyruvoyl-dependent cofactor in ADC and other pyruvoyl-dependent enzymes from the zymogenic form (termed the π-chain). The β-hydroxyl of Ser25 attacks the carbonyl of the previous amino-acid residue (i) to form an oxyoxazolidine intermediate, which decomposes (ii) to form an ester intermediate. E1cB elimination (iii) forms a N-terminal dehydroalanyl residue which is hydrolysed (iv) to form the pyruvoyl cofactor (the α-chain). The ester intermediate can also be hydrolysed to yield the inactive α′-chain (v). (*c*) Schematic of the ADC active site based on the structure of the WT zymogen reported by Schmitzberger *et al.* (2003 ▶). The β-hydroxyl group forms a hydrogen bond to the side-chain amide of Asn72, and the backbone carbonyl of Gly24 forms a hydrogen bond to the β-hydroxyl of Thr57. The phenolic hydroxyls of both Tyr58 and Tyr22 (not shown) are candidates to act as a base to deprotonate the hydroxyl of Ser25.

In bacteria, the biosynthetic pathway for pantothenate consists of four enzymes leading from l-aspartate and α-ketoisovalerate (2-­oxo-­4-methylbutyrate), an intermediate in valine biosynthesis, to d-­pantothenate (Fig. 1 ▶ *a*; Webb *et al.*, 2004 ▶). While the synthesis of pantothenate is limited to eubacteria, archaea, higher plants and fungi, pantothenate is used in all organisms to form both coenzyme A and the phosphopantetheine prosthetic group of acyl-carrier proteins. The absence of the pathway in animals makes the pathway a potential target both for antibacterial chemotherapy (Sambandamurthy *et al.*, 2002 ▶) and herbicide development, as well as for engineering to enhance vitamin production (Webb & Smith, 2011 ▶).

Aspartate α-decarboxylase (ADC) is responsible for the decarboxylation of l-aspartate to form β-alanine in the bacterial biosynthetic pathway (Webb *et al.*, 2004 ▶). The further condensation of β-­alanine with d-pantoate to generate pantothenate is catalysed by pantothenate synthetase. ADC was first purified by Williamson & Brown (1979 ▶), who determined that the enzyme was pyruvoyl-dependent rather than pyridoxal-dependent. The enzyme catalyses the decarboxyl­ation of aspartate *via* the formation of an imine link between the α-amino group of the amino acid and an N-terminal pyruvoyl group (Ramjee *et al.*, 1997 ▶). The conjugated α-iminoamide thus formed can stabilize the nitrogen ylid formed following decarboxylation as an extended vinylagous β-azaenolate. Stereospecific reprotonation, attributed to Tyr58 by Saldanha *et al.* (2001 ▶), then leads to the formation of β-alanine with overall retention of configuration.

Several other pyruvoyl-dependent decarboxylases have been described, including arginine decarboxylase (PDB entry 1n2m; Tolbert *et al.*, 2003 ▶), histidine decarboxylase (PDB entry 1pya; Gallagher *et al.*, 1993 ▶), *S*-adenosylmethionine decarboxylase (PDB entry 1tmi; Toms *et al.*, 2004 ▶) and phosphatidylserine decarboxylase (Schuiki & Daum, 2009 ▶). In all cases the pyruvoyl group is formed from an internal serine residue (Ser25 in ADC) in the zymogen (termed the π-chain) which rearranges to form the N-terminal pyruvoyl group *via* an ester intermediate analogous to the thioester intermediate observed in intein processing (Fig. 1 ▶ *b*; von Poelje & Snell, 1990 ▶). In the case of ADC, this ester intermediate was observed directly in the X-ray structure determined by Albert *et al.* (1998 ▶). The activated protein therefore consists of two chains, the N-terminal β-­chain and the C-terminal α-chain, which includes the N-terminal pyruvoyl group formed from Ser25. In the case of ADC, hydrolysis to yield an N-terminal serine (then termed the α′-chain) is also observed (Ramjee *et al.*, 1997 ▶). The structural basis for formation of the pyruvoyl group was further investigated by Schmitzberger *et al.* (2003 ▶), who reported the crystal structures of both the wild-type (WT) zymogen (shown schematically in Fig. 1 ▶ *c*), the constitutively inactive S25A zymogen and a variety of other site-directed mutants. More recently, we have reported the structure of the N72A site-directed mutant and demonstrated that this residue is not required for activation (Webb *et al.*, 2012 ▶).

The structure of the WT zymogen led Schmitzberger *et al.* (2003 ▶) to propose a role for residue Thr57 in an autocatalytic activation reaction. This residue was proposed to stabilize the zwitterionic 2-oxyoxazolidinyl intermediate formed during ester formation *via* a hydrogen bond from the oxyanion to the β-hydroxyl group of Thr57; this interaction was observed in the structure of the zymogen. Albert *et al.* (1998 ▶) previously proposed that the adjacent residue, Tyr58, might also be involved in the activation of ADC. More recently, we and others have independently reported that the activation reaction is not in fact autocatalytic (Nozaki *et al.*, 2012 ▶; Stuecker *et al.*, 2012 ▶). A second protein, PanZ (also known as PanM), is required for activation *in vivo*. In the absence of this protein, the expressed protein is isolated as the inactive zymogen. In this work, we investigate the effect of mutation of other residues expressed in a *panZ* ^+^ background and report the structure of the site-directed mutant T57V. In this manuscript, we demonstrate that this protein is constitutively inactive; we have subsequently used this observation to characterize the interaction of ADC with PanZ as described elsewhere (Monteiro *et al.*, 2012 ▶)

## 2. Materials and methods   

### 2.1. Generation of site-directed mutants   

The site-directed mutants Y22F, W47A, T57V, Y58F, I60A, S70A and I86A were generated using the QuikChange mutagenesis protocol (Stratagene) with *Escherichia coli panD* subcloned into the expression vector pRSETA as a template together with the following oligonucleotides: Y22F, Y22FF (5-′GCGGACCTGCACTTTGAAGGTTCTTGCGCC-3′) and Y22FR (5′-GGCGCAAGAACCTTCAAAGTGCAGGTC­CGC-3′); W47A, W47AF (5′-GCCATTGATATCGCGAATGTCACCAACGGC-3′) and W47AR (5′-GCCGTTGGTGACATTCGCGATATCAATGGC-3′); T57V, T57VF (5′-GGCAAGCGTTTCTCCGTTTATGCCATCGCG-3′) and T57VR (5′-CGCGATGGCATAAACGGAGAAACGCTTGCC-3′); I60A, I60AF (5′-CCACTTATGCCGCCGCGGCAGAACGCGG-3′) and I60AR (5′-CCGCGTTCTGCCGCGGCGGCATAAGTGG-3′); S70A, S70AF (5′-CGAGAATTATTGCTGTTAACGGTGCGGCGGC-3′) and S70AR (5′-GCCGCCGCACCGTTAACAGCAATAATTCTCG-3′); I86A, I86AF (5′-GGCGATATTGTCGCCATCGCCAGCTTCG-3′) and I86AR (5′-CGAAGCTGGCGATGGCGACAATATCGCC-3′). The site-directed mutant Y58F was generated using overlap PCR using the specific primers Y58FF (5′-ACGTTTGCCATCGCGGCAGAACGCGGTTCG-3′) and Y58FR (5′-GTTGCCGTTCGCAAAGAGGTGCAAACGGTAGCGCCGTCT-3′) as described in Schmitzberger *et al.* (2003 ▶). The DNA sequences of the mutated plasmids were confirmed by DNA sequencing. Proteins were overexpressed and purified as described previously (Schmitzberger *et al.*, 2003 ▶). Protein identity was confirmed by MALDI–TOF mass spectrometry as described previously (Webb *et al.*, 2003 ▶) and protein activation was analysed by Tris–tricine SDS–PAGE and mass spectrometry.

### 2.2. Crystallization, data collection and processing   

Crystallization trials were carried out at 17°C in hanging drops by vapour diffusion with a 1:1 ratio of protein to precipitant. Trials were conducted using Hampton Research Grid Screen Ammonium Sulfate and Grid Screen Sodium Malonate, using a range of protein concentrations from 3 to 15 mg ml^−1^. Crystals were obtained in 1.5, 2.9 and 3.4 *M* sodium malonate pH 4 using a final protein concentration of 7.5 mg ml^−1^. Crystals were cryoprotected using 3.4 *M* sodium malonate pH 4.0. Crystals were mounted in nylon loops (Hampton Research) and flash-cooled in liquid nitrogen. Diffraction data for the T57V mutant were collected on beamline ID14-4 at the European Synchrotron Radiation Facility (ESRF).

### 2.3. Crystal structure refinement   

The diffraction data were indexed, integrated and scaled using *MOSFLM* (Leslie, 2006 ▶) and *SCALA* (Evans, 2006 ▶), and a complete list of structure factors with *R*_free_ flags was generated using the *CCP*4 suite (Winn *et al.*, 2011 ▶). The *R*_free_ set was comprised of a random selection of 5% of the data and was excluded from the refinement. The structure was solved in space group *P*6_1_22 with *MOLREP* (Vagin & Teplyakov, 2010 ▶) using the crystal structure of the native precursor of pyruvoyl-dependent l-aspartate α-decarboxylase (PDB entry 1ppy; Schmitzberger *et al.*, 2003 ▶) as a molecular-replacement model. Refinement was performed using *REFMAC*5 (Murshudov *et al.*, 2011 ▶), riding H atoms were included in the refinement and *B* factors were refined isotropically. Model building was performed after each round of refinement using *Coot* (Emsley *et al.*, 2010 ▶).

## 3. Results and discussion   

### 3.1. Thr57 is required for the post-translational activation of ADC   

The crystal structure of the zymogen of ADC, in which chain cleavage to form the pyruvoyl group has not yet occurred, was determined by Schmitzberger *et al.* (2003 ▶). In this work, it was suggested that the carbonyl of Gly24 forms an essential hydrogen bond to the β-hydroxyl group of the conserved residue Thr57, and that this residue was therefore required for activation (Fig. 1 ▶ *c*). Circumstantial evidence for the requirement for Thr57 was provided by the observed activation state of the insertion mutant S25a-A25b, in which an additional alanine residue is inserted between Ser25 and Cys26 (Schmitzberger *et al.*, 2003 ▶). In the case of S25a-A25b, the inserted Ala25b occupies the position occupied by Ser25 in the WT zymogen structure, and the carbonyl of Ser25a can therefore interact with Thr57 in place of the carbonyl of Gly24. No activation was observed in this site-directed mutant. In contrast, in the G24a-A24b mutant an alanine was inserted between Gly24 and Ser25. This leads to an increase in the size of the flexible loop while leaving the carbonyl of Ser25 to interact with Thr57, and in this case trace activation was observed after overexpression. At this time, the requirement for PanZ in activation had not yet been demonstrated; however, all protein expression was carried out in *panZ* ^+^ *E. coli* strains. The observation of active protein after overexpression but before purification and subsequent incubation therefore cannot determine whether a particular residue is required for either autocatalytic or PanZ-catalysed activation.

**Figure 2:**
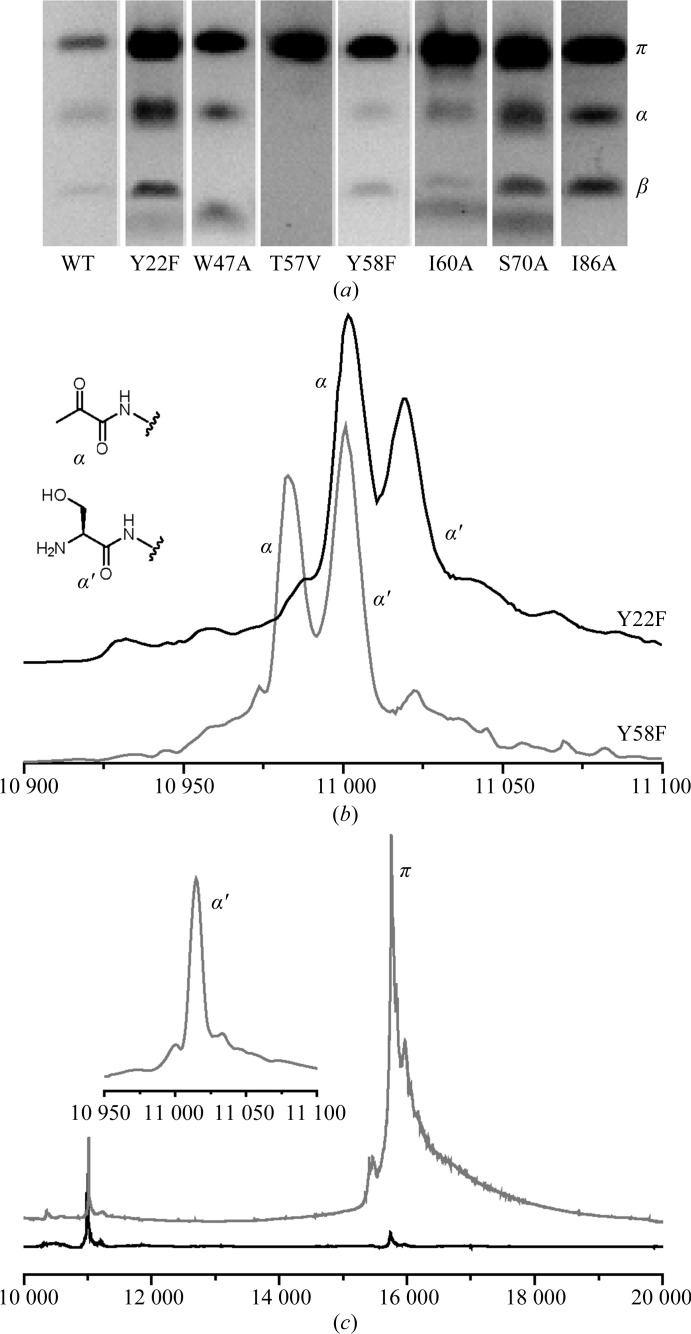
(*a*) 10% Tris–tricine SDS–PAGE analysis of ADC site-directed mutants after purification. Bands corresponding to the zymogen (π, ∼15.5 kDa) and the activated α-chains (∼11 kDa) and β-chains (∼4.5 kDa) are evident for all site-directed mutants other than T57V. (*b*) MALDI–TOF analysis of the Y22F (black) and Y58F (grey) site-directed mutants show two peaks at ∼11 kDa corresponding to the α- and α′-chains, indicating that pyruvoyl cofactor formation occurs in both cases. (*c*) MALDI–TOF analysis of T57V after prolonged incubation at 70°C reveals trace cleavage of the protein to generate only an α′-chain with an N-terminal serine (11 015 Da; grey trace). The proportion of α′-chain relative to π-chain (∼15.5 kDa) is very low compared with other site-directed mutants, which are completely activated after incubation at 37°C (*e.g.* the black trace for Y58F)

We constructed several site-directed mutants of *E. coli* ADC to confirm the role of Thr57 in activation and to investigate the role of other conserved residues close to the active site, including Tyr22, Trp47, Tyr58, Ile60, Ser70 and Ile86. Tyr58 has previously been implicated in the activation of ADC, and it was suggested that this residue acts to deprotonate the Ser25 β-hydroxyl (Albert *et al.*, 1998 ▶). A total of seven different site-directed mutants were overexpressed and purified and their activation states were determined by gel electrophoresis (Fig. 2 ▶). After overexpression in *E. coli* C41 (DE3), purified His-tagged WT ADC is obtained as a mixture of the zymogen and the activated protein. This can readily be determined by the presence of three bands on SDS–PAGE gels at approximately 16, 11 and 4.5 kDa, which correspond to the zymogen (the π-chain), the C-­terminal α-chain (bearing the pyruvoyl group) and the N-terminal β-chain. Three bands were observed for all site-directed mutants other than T57V. The site-directed mutants Y22F and Y58F were further analysed by MALDI–TOF MS (Fig. 2 ▶ *b*). In both cases, peaks corresponding to the π-chain zymogen, the β-chain and both the α- and α′-chains (the hydrolysis product) could be detected, demonstrating that neither residue is required for formation of the pyruvoyl group. This demonstrates that neither Tyr58 nor Tyr22 is required for the activation reaction.

Initial analysis suggested that Thr57 is required for activation, as the T57V mutant cannot be catalytically activated in *E. coli* at 37°C. Ramjee *et al.* (1997 ▶) observed an increased rate of autocatalytic activation of purified ADC at higher temperatures. To confirm that the T57V mutant is incapable of either autocatalytic activation (or catalytic activation by trace co-purified PanZ), the purified protein was incubated at 37 and 70°C for 3 d and re-analysed by SDS–PAGE (data not shown). No activation could be detected by PAGE analysis; however, a small amount of the α′- and β-chains could be detected by mass spectroscopy (Fig. 2 ▶ *c*). The proportion of these products is very low compared with other proteins analysed, and contamination of the mutant with WT protein during growth cannot be ruled out. These observations support the hypothesis that Thr57 is required for the first stage of the activation reaction. The absence of a band corresponding to the α-chain in this sample supports the hypothesis that Thr57 might also be required for controlled cleavage of the ester intermediate, as proposed by Schmitzberger *et al.* (2003 ▶), in a manner analogous to His243 in human *S*-adenosylmethionine decarboxylase (Ekstrom *et al.*, 2001 ▶).

### 3.2. Structural consequences of mutation of Thr57   

To further investigate the role of Thr57 in activation, we determined the three-dimensional structure of the T57V mutant. Schmitzberger and coworkers have previously observed that single-point mutations can lead to substantial rearrangement of the peptide backbone. In the mutant S25A, loss of the hydrogen-bonding interaction between the carbonyl of Gly24 and the β-hydroxyl of Thr57 leads to a 180° rotation of the peptide backbone such that a hydrogen-bonding interaction occurs between the carbonyl of Gly24 and the side-chain amide of Asn72. In the WT zymogen, this residue instead forms a hydrogen bond to the hydroxyl of Ser25. The deletion of the hydrogen-bonding interaction between Thr57 and the carbonyl of Gly24 may lead to a similar structural rearrangement. Alternatively, if Thr57 were only required for cleavage of the ester intermediate then the structure of this intermediate might be resolved, as was the case for the *S*-adenosylmethionine decarboxylase H243A mutant (Ekstrom *et al.*, 2001 ▶).

**Figure 3:**
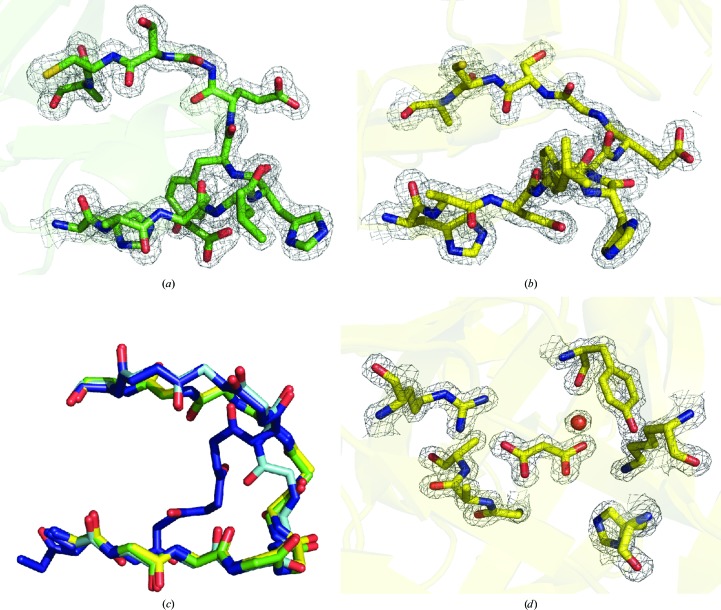
Active-site geometry of the T57V site-directed mutant of ADC. The protein is isostructural to WT ADC (PDB entry 1aw8; Albert *et al.*, 1998 ▶) except in the region of the active site. Electron density is shown as a 2*F*_o_ − *F*_c_ map contoured at 1σ within 1.6 Å. (*a*, *b*) Electron density for the backbone from His19 to Cys26 is well defined in both protomers of the asymmetric unit. Electron density is evident for the β-hydroxyl of Ser25 and there is no evidence for the presence of an ester in the structure. (*c*) Overlay of the backbone structure for the two protomers in the asymmetric unit of the T57V mutant with the two protomers from the unprocessed WT protein (green, T57V protomer *A*; yellow, T57V protomer *B*; blue, WT protomer *A*; cyan, WT protomer *B*). Both protomers adopt a similar conformation distinct from either of the observed conformations in the unprocessed WT structure (PDB entry 1ppy; Schmitzberger *et al.*, 2003 ▶). (*d*) Displacement of the unprocessed chain from the position observed in the unprocessed WT structure can be attributed to binding of malonate to Arg54 in the active site of the mutant. A bound water molecule is illustrated as a red sphere.

The T57V mutant ADC crystallized in space group *P*6_1_22 under similar conditions to those used in previous studies (Schmitzberger *et al.*, 2003 ▶) and was subsequently cryoprotected using sodium malonate. A summary of the crystallographic data statistics for the final model is shown in Table 1 ▶. The backbone is well ordered and the density map is of sufficient quality to allow the structure to be determined for both protomers in the asymmetric unit (Figs. 3 ▶ *a* and 3 ▶ *b*). The structure of the T57V mutant is highly isostructural to the WT zymogen (average r.m.s.d. of 1.742 Å over all atoms) described by Schmitzberger and coworkers, with the exception of the loop region between His17 and Cys26 (Fig. 3 ▶ *c*; average r.m.s.d. of 4.3 Å over all atoms). In this region, the structures of both protomers are similar to each other but in both cases are distinct from the structure observed for the WT zymogen. In the T57V mutant the unprocessed chain is displaced from the active site owing to the binding of a single molecule of the cryoprotectant malonate (Fig. 3 ▶ *d*), which forms ionic interactions with both Lys9 and Arg54. In the crystal structure of ADC from *Helicobacter pylori* (PDB entry 1uhd) described by Lee & Suh (2004 ▶), Arg54 interacts with the β-carboxylate of isoasparagine in line with the model for substrate binding described by Saldanha *et al.* (2001 ▶). This adventitious interaction of malonate in T57V means that the loss of the hydrogen bond between the carbonyl of Gly24 and the β-hydroxyl of Thr57 leads to large-scale rearrangement of the backbone relative to that observed in the WT zymogen. The hydrogen-bond interaction observed between Ser25 and the β-amide of Asn72 in the WT zymogen is lost owing to these changes in the torsional angles of the chain.

### 3.3. Proposed role for Thr57 in the activation of ADC and comparison with other pyruvoyl-dependent enzymes   

On the basis of the crystal structure of the unprocessed WT zymogen, Schmitzberger and coworkers proposed two potential roles for Thr57 in the activation of ADC. The first is that it acts as a general acid to support the formation of the ester intermediate observed by Albert and coworkers by supporting the formation of the negative charge in the oxyoxazolidine intermediate; the second role is that after formation of the ester intermediate it acts as a general base to deprotonate the α-proton of Ser25, leading to chain cleavage and the formation of a dehydroalanine residue. Mutation of Thr57 leads to abolition of the activation reaction at 37°C. This provides direct evidence for its involvement in the first step of the activation reaction: formation of an ester intermediate. The presence of bound malonate in the crystal structure leads to displacement of the unprocessed chain. This means that it is not possible to determine whether the observed conformation in the zymogen is owing to the hydrogen bond between Thr57 and the carbonyl of Gly24 (Schmitzberger *et al.*, 2003 ▶), but it may be that the very lack of this hydrogen bond is critical in allowing malonate to bind. After prolonged incubation at elevated temperatures a small amount of serinolysis can be observed in the T57V mutant by mass spectrometry (Fig. 2 ▶ *b*), but catalytic turnover could not be detected (data not shown), suggesting that this residue may also required for chemoselective cleavage of the ester intermediate to form the pyruvoyl group.

A structural analysis of the activation of arginine decarboxylase has also been undertaken. The structure of an S53A zymogen has been reported (PDB entry 1n2m; Tolbert, Graham *et al.*, 2003 ▶) and the function of two residues, Asn47 and Glu109, in the activation process has been investigated (Soriano *et al.*, 2008 ▶). The N47A site-directed mutant showed a 500-fold lower catalytic activity than the WT enzyme and the crystal structure of this mutant (PDB entry 2qqd) revealed a mixture of activation states, including the structure of the unactivated protein. The side chain of Asn47 is positioned such that it can act as a hydrogen-bond donor to the carbonyl of Ser52 and thereby support the formation of the oxyoxazolidine intermediate in the activation reaction in a manner analogous to Thr57 in ADC.

In human pyruvoyl-dependent *S*-adenosylmethionine decarboxylase (S-AdoMetDC), rearrangement of Ser68 is required to form the pyruvoyl group. The neighbouring residue Ser229 has been shown to be required for activation; site-directed mutation of this residue to alanine yielded an enzyme which could not be activated (Xiong & Pegg, 1999 ▶). In contrast, an H243A mutant showed a decreased rate of activation, but addition of hydroxylamine to the protein led to chain cleavage, demonstrating that His243 is required for the second step of cofactor generation in this enzyme. This was subsequently confirmed by refinement of the structure of this ester intermediate (PDB entry 1jl0, Ekstrom *et al.*, 2001 ▶). Although the structure of an S68A mutant of the human enzyme has been reported (PDB entry 1msv; Tolbert, Zhang *et al.*, 2003 ▶), the structure of the unprocessed form of the human WT enzyme has not been determined. Toms *et al.* (2004 ▶) reported the structure of both the analogous S63A mutant (PDB entry 1tmi) and the unprocessed WT enzyme from *Thermotoga maritima* (PDB entry 1tlu). Unlike the case of ADC, for which the unprocessed WT (PDB entry 1ppy) and S25A mutants show distinct conformations, the two structures are similar in the region of the cleaved chain. In the *T. maritima* S-AdometDC structure, Ser55 (analogous to Ser229 in the human protein) is positioned on the opposite face of the cleaved carbonyl group to the hydroxyl of Ser63 and would appear to be able to play a similar role to Thr57 in ADC; however, the S55A mutant of the *T. maritima* protein was still activated. In this protein, residue Glu62 can act as a general base to deprotonate Ser63 and thereby promote formation of the oxy­oxazolidine intermediate; it is possible therefore that the presence of only one of the two catalytic residues is sufficient to promote the activation reaction in this system.

## 4. Conclusion   

In conclusion, we have screened the effect of mutation at several conserved residues proximal to the active site of ADC, including Tyr22, Thr57 and Tyr58. The latter two residues have previously been implicated in the activation reaction by Schmitzberger *et al.* (2003 ▶) and Albert *et al.* (1998 ▶), respectively. We conclude that only Thr57 is required for activation. The absence of a residue in PanD that is capable of deprotonating Ser25, as would be expected for autocatalysis, suggests a possible role for PanZ in the catalysed reaction. A structural analysis of the zymogen reveals that substantial backbone rearrangement occurs as a result of the adventitious binding of a molecule of malonate in the region of the unformed active site; however, there is no evidence for ester formation in the protein, suggesting that Thr57 is required for the formation of the ester intermediate. Prolonged incubation at high temperatures leads to trace serinolysis of the protein, suggesting that Thr57 is also required for the second step in activation: the specific chain cleavage to form the dehydroalanyl residue from which the pyruvoyl cofactor is formed. The identification of this constitutively inactive mutant of ADC provides a key resource to understand the activation reaction, and we have already reported its use in characterizing the interaction between PanD and PanZ (Monteiro *et al.*, 2012 ▶).

## Supplementary Material

PDB reference: aspartate decarboxylase, T57V mutant, 4azd

## Figures and Tables

**Table 1:** Data-collection and refinement statistics Values in parentheses are for the outermost shell of the resolution range.

Data collection	
Beamline	ID14-4, ESRF
Wavelength (Å)	0.9795
Temperature (K)	100
Space group	*P*6_1_22
Unit-cell parameters (Å, °)	*a* = 69.9, *c* = 217.7, α = β = 90, γ = 120
Resolution (Å)	26.46–1.62 (1.67–1.62)
*R*_merge_	0.08 (0.48)
〈*I*〉/σ(〈*I*〉)	7.4 (1.4)
Completeness (%)	97.8 (99.9)
Wilson *B* (Å^2^)	16.1
Multiplicity	4.4 (4.3)
No. of reflections	167294
No. of unique reflections	37850
Refinement	
*R* factor	0.185 (0.329)
*R*_free_	0.217 (0.361)
No. of atoms	
Protein	1833
Ligands	28
Water	138
Average *B* factors (Å^2^)	
Protein	13.5
Malonate	16.2
Waters	23.2
R.m.s. deviations	
Bond lengths (Å)	0.028
Bond angles (°)	2.72
Ramachandran statistics (%)	
Most favoured	97.6
Generously allowed	2.0
Disallowed	0.4
PDB code	4azd
